# Urinary aberrations in systemic lupus erythematosus not always indicative of lupus nephritis: a cross-sectional cohort study

**DOI:** 10.1007/s10067-023-06682-w

**Published:** 2023-07-13

**Authors:** Linnea Karlsson, Agneta Zickert, Elisabet Svenungsson, Jan Schmidt-Mende, Francesca Faustini, Iva Gunnarsson

**Affiliations:** 1 Department of Medicine, Division of Rheumatology, Karolinska Institutet, Solna, Sweden; 2 Rheumatology, Karolinska University Hospital, Stockholm, Sweden; 3 Department of Clinical Pathology and Cytology, Karolinska University Hospital, Stockholm, Sweden

**Keywords:** Histopathology, Kidney biopsy, Lupus nephritis

## Abstract

**Introduction:**

Kidney biopsy is the reference tool for diagnosing and guiding treatment strategies in inflammatory renal diseases, such as lupus nephritis (LN).

We investigated the histopathological findings in first-time kidney biopsies from a large cohort of SLE patients. We focused on the occurrence and type of histopathological findings other than LN, and fulfillment of renal criteria in established SLE classification systems were analyzed.

**Methods:**

We retrospectively included SLE patients (*n* = 139) who underwent a first kidney biopsy between 1995 and 2021, upon clinical suspicion of renal involvement. Based on histology, two groups were defined, LN and non-LN, for which clinical and laboratory features were compared.

**Results:**

Findings consistent with LN according to ISN/RPS classification system were present in 123/139 patients (88.5%) and findings not consistent with LN were present in 16 /139 (11.5%).

Non-LN patients were older at SLE diagnosis compared to LN patients (M, years 38.0 vs. 30.1, *p*=0.013) and had longer disease duration (M, years 11.9 vs 0.5) (*p*=0.027).

Among non-LN patients 85.7% met the SLICC criteria item for renal SLE, seen in 94.7% in the LN group (ns). For the ACR/EULAR criteria, 66.7% of the non-LN group fulfilled the criteria compared to 74.8% in LN patients (ns). Proteinuria below the criteria cut-off level (< 0.5 g/24 h) was seen in 20% of patients with class III/IV LN.

**Conclusion:**

Our data confirm the importance of kidney biopsy for ruling out the presence of renal pathology other than LN. Patients with low-grade proteinuria may exhibit severe types of LN, which reinforces the need for early biopsies to detect LN.

**Table:** 

**Key Points** • *Our findings show that histopathology changes other than lupus nephritis may occur in a significant number of patients with clinical and laboratory signs of novel kidney involvement.* • *Low-grade proteinuria does not exclude findings of active lupus nephritis that require the start of immunosuppressive therapy.* • *The study stresses the importance of performing kidney biopsies also in the presence of low-grade proteinuria or when signs of kidney function abnormalities occur.* • *This is crucial as early detection and prompt initiation of therapy may improve outcomes in lupus nephritis.*

## Introduction

Kidney biopsy is the gold standard for diagnosing lupus nephritis (LN), a major organ involvement in systemic lupus erythematosus (SLE). It allows the estimation of the type and severity of LN and helps in treatment decision-making. Also, it enables ruling out other conditions that may mimic the clinical picture of LN [1]. According to the American College of Rheumatology (ACR) current recommendations, kidney biopsy should be performed in case of significant proteinuria, and/or haematuria, or in case of otherwise unexplained deterioration of renal function [2]. The joint European Alliance of Associations for Rheumatology/ European Renal Association-European Dialysis and Transplant Association (EULAR/ERA-EDTA) recommendations are less strict and suggest to perform a kidney biopsy at “any sign of renal involvement” in SLE patients, aside from mandatory cases of proteinuria ≥ 0.5 g/24 h with or without haematuria or cellular casts in urine sediment [3]. The presence of proteinuria or red blood cell (RBC) casts in urine sediment are the core items for the clinical definition of renal involvement for the commonly used SLE International Collaborating Clinics (SLICC) classification criteria [4], while the more recent 2019 EULAR/ACR criteria consider only proteinuria [5].

Performing kidney biopsy according to the indications established in current recommendations aims at confirming LN by histological assessment. To what extent other histological findings not consistent with LN occur in the initial suspicion of LN has however not been fully explored.

In a large SLE population, we studied the histopathology in first-time kidney biopsies, performed due to suspicion of LN. Our aim was to quantify the occurrence of non-LN-related findings and to compare clinical and laboratory features, and fulfillment of classification criteria, of patients with biopsy-confirmed LN and those with non-LN-related findings.

## Patients and methods

Patients from the Karolinska SLE cohort who had a first-time suspicion of renal involvement between 1995 and 2021 and underwent kidney biopsy were included. All patients fulfilled the ACR 1982 and/or SLICC disease classification criteria for SLE [4, 6] and were biopsied on clinical indication.

Demographic and clinical data were collected from electronic medical charts. All patients had given written informed consent to participate, and the study was undertaken in accordance with the principles of the Declaration of Helsinki.

### Laboratory variables

Laboratory investigations included urinalysis (dipstick) and urine sediment and determination of albuminuria, either on 24-h urine collection or as albumin-to-creatinine ratio (u-ACR), depending on the routine methods available at the time of biopsy. To align with the thresholds of proteinuria used in classification criteria and recommendations [4, 5], we used equivalent thresholds of albuminuria/24-h and u-ACR according to the conversion methods described [7, 8]. Active urine sediment was defined as the presence of haematuria (> 5 RBCs per high-power field) in urine sediment.

Renal function was estimated by plasma creatinine (µmol/L) and estimated glomerular filtration rate (eGFR) (mL/min/1.7 m^2^) using the Lund-Malmö equation (LM-revised) [9].

Immunological analyses included anti-dsDNA antibody and complement (C3 and C4) levels measurements. Since routine methods have changed over the years, we handled the results as dichotomous variables (positive/negative for anti-dsDNA and decreased/not decreased for complement). Anti-neutrophil cytoplasmic antibodies (ANCA) were analyzed either by immunofluorescence displaying a perinuclear (p-ANCA) or cytoplasmic (c-ANCA) pattern, or by ELISA with detection of antibodies against proteinase 3 (PR3) or myeloperoxidase (MPO). All immunological analyses were performed according to the clinical routine at the department of immunology at the hospital.

### Evaluation of disease activity

Disease activity was measured by the SLE disease activity index 2000 (SLEDAI-2 K) at the time of kidney biopsy [10]. For statistical analysis, we also calculated the renal component with only grading or urine variables as a renal SLEDAI (rSLEDAI).

### Histopathology

All biopsies were evaluated through light microscopy, immunofluorescence, and electron microscopy, by experienced pathologists and classified according to the International Society of Nephrology/Renal Pathology Society (ISN/RPS) criteria for LN [11]. Findings not consistent with LN were described and classified according to standardized histopathological definitions.

### Statistics

Continuous variables are described as the median and interquartile range (M, IQR) after testing for normal distribution. Categorical variables are presented as numbers and percentages. Non-parametric statistic tests were applied as appropriate to compare medians (Mann-Whitney U-test) and the frequency of categorical variables (Fisher’s exact test or chi-square test) between groups. *p*-values < 0.05 were considered statistically significant.

## Results

Briefly, 139 patients (76.3% females) with a median (IQR) age of 34.5 (26.1–52.4) years were included. At the time of the kidney biopsy, the majority of the patients were receiving oral corticosteroids (69.4%), and 18.2% were on immunosuppressants. Table 1 summarizes the demographic and clinical characteristics of the study patients.

**Table 1:** Clinical and demographic characteristics of the study patients

	All (*n* = 139)	LN (*n* = 123)	Non-LN (*n* = 16)	*P*-value
Female; *n* (%)	106 (76.3)	93 (75.6)	13 (81.3)	0.762
Age at SLE diagnosis (years); M (IQR)	32.2 (22.8–44.6)	30.1 (21.9–41.5)	38.0 (33.3–51.1)	**0.013**
Age at SLE biopsy (years); M (IQR)	34.5 (26.1–52.4)	32.9 (25.3–50.1)	55.1 (49.5–64.4)	** < 0.001**
SLE duration at biopsy (years); M (IQR)	0.6 (0.0–5.3)	0.5 (0.0–4.5)	11.9 (0.1–25.8)	**0.027**
Systolic blood pressure (mm Hg); M (IQR)	123.0 (113.0–140.0)	120.0 (111.0–140.0)	140.0 (115.5–153.8)	0.101
Diastolic blood pressure (mm Hg); M (IQR)	80.0 (70.0–85.0)	80.0 (70.0–85.0)	80.0 (71.0–94.3)	0.136
Patients on ACEi and/or ARB; *n* (%)*	29/126 (23.0)	25/111 (22.5)	4/15 (26.7)	0.747
Patients on Prednisone; *n* (%)*	83/120 (69.4)	73/105 (69.5)	10/15 (66.7)	0.775
Prednisone equivalent dose (mg/day); M (IQR)	10.0 (0–20.0)	10 (0.0–20.0)	10.0 (0.0–40.0)	0.961
Patients on DMARDs; *n* (%)*	24/132 (18.2)	18/116 (15.5)	6/16 (37.5)	0.076
Ethnicity				0.658
Caucasian; *n* (%)	118 (84.9)	102 (82.3)	16 (100.0)	–
Asian; *n* (%)	8 (5.8)	8 (6.5)	0	–
African; *n* (%)	7 (5.0)	7 (5.7)	0	–
Hispanic; *n* (%)	6 (4.3)	6 (4.9)	0	–
P-Creatinine (µmol/L); M(IQR)	72.0 (61.0–91.0)	70.0 (60.0–88.0)	78.5 (68.5–141.3)	**0.038**
eGFR at baseline; M (IQR)	87.0 (68.6–98.3)	88.3 (72.1–100.3)	67.9 (31.2–89.3)	**0.003**
Proteinuria ≥ 0.5 g/24 h or u-ACR ≥ 30 mg/mmol; *n* (%)*	91/123 (74.6)	83/111 (75.5)	8/12 (66.7)	0.342
Active urine sediment; *n* (%)*	92/117 (78.6)	82/104 (78.8)	10/13 (76.9)	1.000
Anti-dsDNA-antibodies; *n* (%)*	96/120 (80.0)	90/107 (84.1)	6/13 (46.2)	**0.004**
Hypocomplementemia † at biopsy time-point; *n* (%)*	75/98 (76.5)	73/90 (81.1)	2/8 (25.0)	**0.002**
SLEDAI total score, M (IQR)	12.0 (8.0–18.0)	12.0 (8.3–18.0)	11.0 (4.5–16.0)	0.144
SLEDAI renal score, M (IQR)	8.0 (4.0–12..0)	8.0 (4.0–12)	8.0 (1.0–12.0)	0.846

*SLE*, systemic lupus erythematosus; *M*, median; *IQR*, interquartile range; *ACEi*, angiotensin converting enzyme inhibitor; *ARB*, angiotensin receptor blocker; *n*, number; *eGFR*, estimated glomerular filtration rate, *hrs*: hours, *u-ACR*: urine albumin-to-creatinine ratio, *dsDNA*, double-stranded DNA; *SLEDAI*, SLE disease activity index

^*^Data missing in a subset of patients

^†^Defined as decreased levels of complement C3 and/or C4

### Histopathological findings

At the evaluation, 123 patients (88.5%) had findings consistent with LN according to the ISN/RPS classification system [11] of whom 25 patients (20.3%) had classes I and II, 41 (33.3%) class III, 31 (25.2%) class IV, and 26 (21.1%) class V.

Sixteen patients (11.5%) did not have changes consistent with LN (Table 1). Seven patients had evidence of pure vasculitis with no signs of LN. Of these, 5/6 patients investigated were found to be positive for either p-ANCA or MPO and were re-diagnosed with concomitant ANCA-associated vasculitis to the SLE diagnosis. For details of the non-LN subset, see Table 3.

### Comparisons between LN and non-LN findings

Patients in the non-LN group were significantly older at the time of SLE diagnosis and at kidney biopsy compared to LN patients and had longer disease duration. Also, their renal function was worse at a group level. Anti-dsDNA antibody positivity and complement consumption were more common among patients with confirmed LN (Table 1). There was no difference in SLEDAI or renal SLEDAI scores at the time of kidney biopsy comparing patients with LN and non-LN findings (for details, see Table 1).

### Proteinuria

Data on proteinuria levels was available in 123 cases, but data was missing in 4 non-LN and 12 LN patients. Among all patients with available data, 91 had proteinuria ≥ 0.5 g/24 h, 83/111 LN and 8/12 non-LN (ns).

Thirty-two patients had proteinuria < 0.5 g/24 h, 28 LN and 4 non-LN. Of the LN patients, 14/28 had classes III and IV LN. The remaining 14 LN cases with low-grade proteinuria consisted of 8 patients with classes I and II (47.1%) and 6 with class V (25%), respectively, of all classes I, II, and V in the study cohort. In total, 14/70 (20%) of the class III/IV LN population had low-grade proteinuria.

Table 2 illustrates the partition of histopathological findings in relation to the fulfillment of clinical definitions of LN according to SLICC and ACR/EULAR criteria.

**Table 2:** Histopathologic findings at kidney biopsy

Diagnosis according to ISN/RPS	*n* (%)	SLICC criteria: proteinuria ≥ 0.5 g/24 h or u-ACR ≥ 30 mg/mmol and/or red blood cell casts in sediment; *n* (%)*	EULAR/ACR criteria: proteinuria ≥ 0.5 g/24 h or u-ACR ≥ 30 mg/mmol; *n* (%)*
LN	123 (88.5)	107/113 (94.7)	83/111 (74.8)
Classes I and II	25 (20.3)	19/20 (95.0)	9/17 (52.9)
Classes III and IV	72 (58.5)	65/68 (95.6)	56/70 (80.0)
Class V	26 (21.1)	23/25 (92.0)	18/24 (75.0)
Non-LN	16 (11.5)	12/14 (85.7)	8/12 (66.7)
Vasculitis	7	6/6	5/6
Hypertensive nephrosclerosis	3	2/3	1/2
aPLN	2	1/2	1/2
IgAn	1	1/1	1/1
TIN	1	–	–
tGBM	1	1/1	0/1
No overt findings	1	1/1	-

*ISN/RPS*, International Society of Nephrology/Renal Pathology Society; *u-ACR*: urine albumin-to-creatinine ratio; *aPLN*, anti-phospholipid assiociated nephropathy; *IgAn*, IgA nephropathy; *TIN*, tubulointerstitial nephropathy; *tGBM*, thin glomerular basement membranes; *n*, number

^*^Data missing in a subset of patients

Data on urine sediment was available in 117 patients. Complete data on proteinuria and/or urine sediment was at hand for 127 patients. Of these, 119 (93.7%) had proteinuria above the threshold level and/or an active urine sediment, thus fulfilling the SLICC clinical definition of LN [4]. The ACR/EULAR clinical criterion for LN was met by 91 (74%) patients [5] (Table 3).

**Table 3:** Characteristics of patients with non-LN histopathology

Non-LN findings in renal tissue	Age, y	Gender	Disease duration	ACR criteria	ANA ever	Anti dsDNA ever	Low C3/C4 ever	Low C3/C4 at biopsy	ANCA at biopsy
Vasculitis	44.7	M	− 3.34*	3	Yes	–	–	–	MPO
Vasculitis	52.0	F	28.3	7	Yes	Yes	Yes	Yes	MPO
Vasculitis	34.2	F	− 0.25 *	7	Yes	No	No	No	p-ANCA IFL
Vasculitis	56.1	F	21.6	5	Yes	No	No	–	–
Vasculitis	32.5	F	0.0	6	Yes	No	No	No	neg
Vasculitis	73.9	F	0.38	6	Yes	–	–	–	p-ANCA IFL
Vasculitis	57.0	F	29.9	6	Yes	No	No	No	MPO
Hypertensive nephrosclerosis	53.8	F	3.27	4	Yes	No	No	No	–
Hypertensive nephrosclerosis	68.2	F	28.9	5	Yes	No	No	–	–
Hypertensive nephrosclerosis	65.7	M	0.0	4	Yes	No	No	No	–
aPLN	54.1	F	20.2	5	Yes	No	No	–	-
aPLN	48.7	F	12.1	6	Yes	–	–	–	neg
IgAn	60.3	M	27.22	4	Yes	–	–	–	–
TIN	67.9	F	14.2	4	Yes	No	No	–	–
tGBM	52.4	F	1.1	8	Yes	No	No	No	neg
No overt findings	56.8	F	11.7	6	Yes	Yes	Yes	Yes	–

*ACR*, American College of Rheumatology; *ANA*, antinuclear antibodies; *ANCA*, antineutrophil cytoplasmic antibodies; *MPO*, myeloperoxidase; *IFL*, immunofluorescence; *aPLN*, anti-phospholipid assiociated nephropathy; *IgAn*, IgA nephropathy; *TIN*, tubulointerstitial nephropathy; *tGBM*, thin glomerular basement membranes

^*^SLE diagnosis established after kidney biopsy time-point

Of the 123 patients with biopsy findings consistent with LN, 107 (94.7%) fulfilled the criteria for LN as defined in the SLICC [4], while only 83 (74.8%) fulfilled the ACR/EULAR criteria for LN [5].

Among the non-LN patients with all urine data available (*n* = 14), the definition of LN according to the SLICC [4] criteria was fulfilled by 12 (85.7%) and 8 (66.7%) according to the ACR/EULAR criteria [5]. The rate of the fulfillment of the clinical definitions of LN according to SLICC and ACR/EULAR was no different between the groups (ns).

## Discussion

In this study, we revised the histological findings in a large cohort of SLE patients undergoing kidney biopsy for the first time upon clinical suspicion of LN. Two major findings emerge from our analysis. First, the occurrence of histopathological findings not consistent with LN was rather high, 11.5%, and these patients fulfilled the classification criteria to the same extent as LN patients [4, 5]. Low-grade proteinuria was present in 20% of the cases with severe LN histotypes (class III/IV).

Patients with non-LN findings were in general older and had longer disease duration compared to pure LN cases which stresses the importance of performing biopsies also in established SLE with new onset of renal abnormalities. Of note, among the non-LN patients, we found a significant number of patients with an overlap between SLE and ANCA-associated vasculitis findings. Of these, despite a verified diagnosis of SLE, a majority also had positive ANCAs either by ELISA or immunofluorescence. Similar findings have previously been shown [12], thus stressing the importance to ascertain a correct histopathological diagnosis in treatment decision-making.

The other relevant aspect we highlight is that active histopathological changes can be present despite limited urinary protein excretion. This, in line with previous studies [13, 14], underlines the relevance of performing biopsies early when urinary abnormalities occur. Detecting LN early allows prompt initiation of treatment in order to preserve nephrons [15] and prevent loss of renal function.

The strength of the study is the real-life approach using a large SLE cohort including all available kidney biopsies performed under suspicion of LN in clinical practice. The retrospective design is a limitation, where we depend on the availability of measurements of renal and immunological parameters and changing laboratory methods over the years. Moreover, according to local routine, we rely on measurements of urine albumin, rather than total proteinuria. We here aligned our measurements to total proteinuria according to available references in the literature [7, 8], which gives a good approximation of proteinuria levels.

In summary, a kidney biopsy is the only tool which can confirm the occurrence of LN and thereby determine the need for immunosuppressive treatment. Urine findings or disease activity could not discriminate between LN and other causes of renal abnormalities; furthermore, severe LN (classes III and IV) occurred in as much as 44% (14/32) of patients with low-grade proteinuria. Thus, biopsies should be performed early and current guidelines for performing kidney biopsies, and the classification criteria for LN seem to be insufficient in clinical practice. Based on our results, we believe that biopsies in the early phase of new-onset urinary abnormalities, followed by rapid initiation of immunosuppressive therapy can decrease the risk for future impairment of renal function. Conversely, ruling out other forms of renal pathology can help avoid initiating inappropriate treatment.

## Data Availability

Data sharing is available on reasonable request.
